# On the Nature of Energy-Feasible Wireless Nanosensor Networks

**DOI:** 10.3390/s18051356

**Published:** 2018-04-27

**Authors:** Sebastian Canovas-Carrasco, Antonio-Javier Garcia-Sanchez, Joan Garcia-Haro

**Affiliations:** Department of Information and Communication Technologies, Technical University of Cartagena, 30202 Cartagena, Spain; sebas.canovas@upct.es (S.C.-C.); joang.haro@upct.es (J.G.-H.)

**Keywords:** energy harvesting, nanodevice, piezoelectric generator, terahertz communications, wireless nanosensor networks

## Abstract

Electromagnetic nanocommunications, understood as the communication between electronic nanoscale devices through electromagnetic waves in the terahertz band, has attracted increasing attention in recent years. In this regard, several solutions have already been proposed. However, many of them do not sufficiently capture the significance of the limitations in nanodevice energy-gathering and storing capacity. In this paper, we address key factors affecting the energy consumption of nanodevices, highlighting the effect of the communication scheme employed. Then, we also examine how nanodevices are powered, focusing on the main parameters governing the powering nanosystem. Different mathematical expressions are derived to analyze the impact of these parameters on its performance. Based on these expressions, the functionality of a nanogenerator is evaluated to gain insight into the conditions under which a wireless nanosensor network (WNSN) is viable from the energetic point of view. The results reveal that a micrometer-sized piezoelectric system in high-lossy environments (exceeding 100 dB/mm) becomes inoperative for transmission distances over 1.5 mm by its inability to harvest and store the amount of energy required to overcome the path loss.

## 1. Introduction

Progress in the area of nanotechnology is pushing for new communication techniques to coordinate numerous nanoscale devices. These so-called nanodevices would be able to gather physical parameters at the nanoscale, monitoring and even acting on scenarios yet unexplored. Nanodevices would enable unimaginable applications in many fields such as medicine (e.g., detecting cancerous cells or targeted drug delivery), environmental science (e.g., measurement of atmosphere components) or industry (e.g., critical nanofractures’ detection in machines or structures). Nevertheless, owing to their limited size, nanodevice resources are severely limited, which hinders their capacity to perform a stand-alone service. Therefore, the ability to carry out useful applications effectively relies on a cooperative effort among nanodevices, establishing wireless nanosensor networks (WNSN).

Scientific literature reveals how electromagnetic (EM) nanodevices should communicate [1,2,3,4,5], proposing innovative communication techniques for media access control, synchronization or addressing which are adapted to the extreme constraints of nanodevices (low computing resources, transmission in the THz band, short communication range or high number of nodes, among others). All these works contribute by motivating and suggesting valuable advances in WNSN. However, there is a major limiting factor to be analyzed more thoroughly, energy management, which has a direct impact on the feasibility of an operational WNSN.

In this regard, authors in [6] proposed the first energy analysis for nanonetworks, introducing a powering nanosystem composed of a piezoelectric nanogenerator and a nanocapacitor. The former converts mechanical energy into electrical while the latter stores the energy generated. Based on this nanosystem, with determined specifications (a size of 1000 µm^2^ for both components; 6 pC per charge cycle for the nanogenerator and 9 nF for the nanocapacitor), authors capture the nanonetwork energetic behavior. To this effect, a probabilistic analysis of the network traffic and interferences between nodes is developed to obtain a distribution of the energy available in each node. The energy consumption assumed in [6] for communication is constant and fixed to 1 pJ per pulse (10 W) for transmission and 0.1 pJ in the reception of a pulse, regardless of the medium in which nodes are deployed.

From a different approach but based on the same powering solution (piezoelectric generator together with a capacitor), this paper aims to investigate the energy scarcity problem in WNSN from a more realistic perspective, analyzing those factors that should be considered in an energy-feasible WNSN. This study is motivated by the high transmission powers that have been assumed by different already published communication schemes [4,7] to overcome the huge path loss at terahertz frequencies when the nanonetwork is deployed in a lossy medium (e.g., the human body). In many cases, these transmission powers might be unaffordable for nanodevices. To this end, we focus on the impact of tuning a set of different factors on the behavior of a nanonetwork, not being constrained to specific values of, for instance, the area or transmission energy treated in isolation, unlike [6]. We also evaluate how the increase in both energy (power) transmission and path loss jeopardize the feasibility of a WNSN. In addition, the influence of different key parameters involved in a WNSN (such as generator and capacitor area, energy source, or particular technology employed) are also analyzed towards the design of a proper and feasible solution depending on the application. In this manner, the paper’s organization and its main contributions can be summarized as follows.

In Section 2, we explore and synthesize the main issues impacting on the energy consumption in WNSN. First, we evaluate the influence of the energy devoted to communication compared to the total energy consumption in a nanodevice. The power transmission values considered have been employed in the related literature. Second, a straightforward communication scheme is proposed to illustrate numerically the number of pulses, and thus the energy consumption, required to conduct a realistic communication between two nanodevices. Finally, we review three conceivable network topologies proposed in diverse works, summarizing their strengths and limitations from the energetic point of view. 

In Section 3, we concentrate upon the most accepted solution to power nanodevices, based on a piezoelectric generator, highlighting its main features. Then, we analyze the three principal factors impacting on the powering nanosystem performance (area, technology, and energy source). To this effect, different mathematical expressions are derived for each factor. Results obtained lead to relevant conclusions to be considered in future nanodevice and WNSN designs.

Section 4 covers the study of the feasibility of WNSN from an energetic point of view, deriving mathematical expressions to link energy consumption with powering factors. The results provide insight into the communication characteristics of feasible EM-based WNSN. Finally, Section 5 concludes the paper.

## 2. Energy Consumption in Wireless NanoSensor Networks (WNSN): Key Factors

There are several factors impacting on the energy consumption of EM-based WNSN, which should be properly tuned depending on the application. In this section, we identify and review the most important, highlighting their main features.

### 2.1. Transmission Power

**Figure 1:**
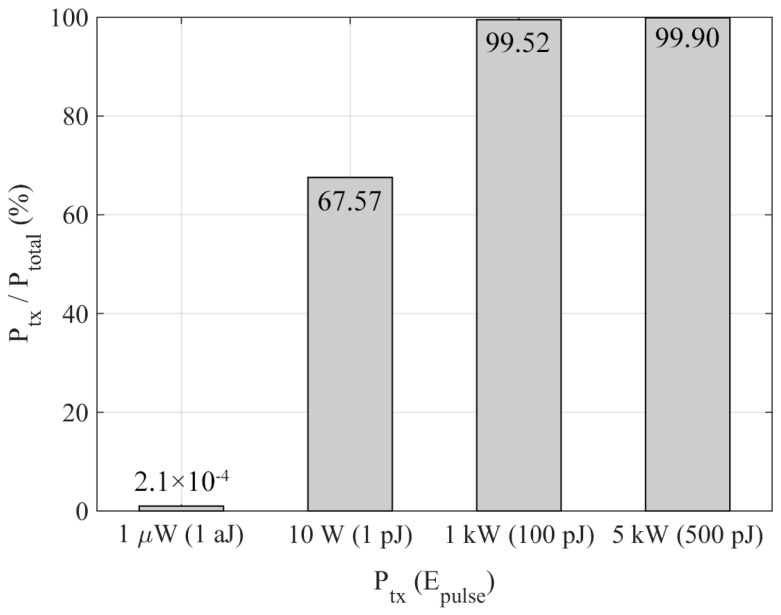
Relation between power required for communication and the total power consumption for different transmission powers.

The dominant communication mechanism in EM-based WNSN is the so-called Time Spread On-Off Keying (TS-OOK) modulation [3], which employs a pulse to transmit a logical “1” and the logical “0” is sent as silence. The time duration of each pulse is set to 100 femtoseconds, although the energy per pulse (*E_pulse_*) varies for each work in the related literature, leading to different transmission powers. These proposed transmission powers (*P_tx_*) fluctuate from 1 µW to 5 kW, that is, from 1 aJ to 500 pJ per pulse [4,5,7,8,9,10,11,12]. Low power values are used for WNSN deployed in low-loss environments (e.g., the air) and micro-range communications (which depends on network topology). Conversely, whether the medium presents high water content (e.g., biological tissues) or the communication range increases (few millimeters), greater power values are required to overcome the high path loss resulting from working at THz radiation frequencies. Although the pulse duration is extremely short, these transmission powers entail significant energy consumption when compared to the energy consumption of the remaining components of the nanodevice denoted as *E_dev_*, which can be expressed as *E_dev_* = *P_dev_ t_on_*, being *P_dev_* the power consumed by the non-communicating parts of the nanodevice when it is active during a period (*t_on_*). For the sake of clarity, in Figure 1, the transmission power is compared to the total nanodevice power consumption (*P_total_*), including all the nanodevice components (processor, memories, buffers, sensor, and radiocommunication system): *P_total_* = *P_tx_* + *P_dev_*. The value of *P_dev_* is estimated to be approximately 240 nW [13].

As can be observed, for a low transmission power the percentage of power with respect to the total is lower than 1%, whereas for a higher transmission power this value rises to 99%. In the latter case, communication among nanodevices poses a real challenge that must be carefully analyzed when developing a communication protocol for WNSN.

### 2.2. Pulses Transmitted with a Single Charge 

As mentioned, the EM communication envisioned for WNSN is based on the exchange of electromagnetic pulses. Thus, the total energy required to conduct the transmission of a particular amount of information (*E_tx_*) is determined by the number of pulses (*n_pulse_*) radiated during the entire packet exchange: *E_tx_* = *E_pulse_ n_pulse_*. As an example, let us suppose a straightforward communication scheme, similar to the protocol proposed in [4], for a WNSN intended to measure blood cholesterol. The communication starts when a nanodevice sends a transmission request packet, containing 24 bits for synchronization, 48 bits devoted to transmitter and receiver addresses, and 8 bits of checksum to detect transmission errors. Upon the acknowledgement packet reception, the transmitter begins the transmission of a data packet. It includes the same fields as the transmission request packet together with the useful data (payload). The number of bits assigned to the payload will ultimately depend on the application for which the WNSN is envisioned. In this case, cholesterol levels in a human body usually range from 20 mg/dL to 300 mg/dL. Thus, in order to cover the entire range with a sensor resolution of 1 mg/dL, the data length must be 9 bits or more, leading to a data packet length of 89 bits.

Thereby, the complete communication consists of 169 bits (80 for the transmission request packet and 89 for the data packet), so the transmitter nanodevice must be able to send at least 85 pulses with one single charge, considering that bits ‘1’ and ‘0’ are equally distributed (probability of sending a pulse (*p_pulse_*) equal to 0.5). Even though this value might seem easily affordable, it is an important concern for nanodevices when the energy per pulse becomes too high, as we will further discuss. We should note that low-weight codes for WNSN have been proposed to optimize the probability of sending a ‘0′ and then reduce the number of pulses transmitted [14]. However, this optimization depends on the channel and network conditions, so for the sake of simplicity, an equitable distribution of ‘1’ and ‘0’ has been considered.

### 2.3. Network Topology

Network topology impacts on two key issues: the distance between nanonodes and the communication techniques required. In this sort of network, the EM path loss reaches huge values due to the absorption loss operating in the THz band. In high-lossy mediums (e.g., blood), path loss can reach values up to 120 dB per millimeter [15]. Thus, the distance between nanodevices belonging to the WNSN is a crucial factor that determines the transmission power needed to ensure an acceptable power density at the receiver. For instance, a small difference of 0.5 mm entails 60 dB of attenuation, which is barely enough to cut off the communication. On the other hand, the communication protocols employed in a WNSN will be subject to the network topology, which directly affects the energy consumption. To illustrate both questions, the most common topologies proposed in the literature for WNSN are reviewed, tackling their routing scheme from the energy consumption viewpoint. Table 1 specifies the main strengths and limitations of each network topology.

**Cluster-based network**: a cluster-based hierarchical architecture was first proposed in [1], and it has been taken as a reference for most WNSN topologies. This topology breaks down the WNSN into different tiers, each one at a different level. The lowest level consists of the smallest and less powerful elements, called nanonodes. All nanonodes are grouped into clusters, merely depending on their location. Each cluster is managed by a larger and less resource-constrained device, denoted as nanorouter or nanocontroller, which directly communicates with its cluster, either through single-hop or a multi-hop communication. Finally, nanorouters are usually attached to the so-called gateway which is entrusted with the connection of the WNSN with a (macro) device, for instance, a smartphone or a computer. Under this routing scheme, the assignment of network addresses can be optimized by dividing them into subranges, each one assigned to one cluster, which would act as a subnanonetwork. This approach shrinks the number of bits required for addressing, thus reducing the energy employed for data transmission. Furthermore, this hierarchy allows the communication protocol to push the network operation complexity towards the nanorouter, releasing nanonodes from this computing load. This topology supports the design of energy-efficient communication schemes in which each nanonode fits the energy required to send a packet by tuning, in each transmission, different communication parameters, such as power transmission or the number of hops to reach the nanorouter [2,9,16]. However, this architecture poses problems when the WNSN is not static, since clusters would change continuously. This variability entails the reconfiguration of the WNSN addressing too frequently, which would result in an excessive waste of energy for nanodevices. Therefore, this topology seems suitable for static WNSN or with limited nanonodes movement (e.g., gathering information about the variation in number and size of cancer cells).

**Mesh network**: mesh topologies for WNSN are planned to cover a unit of area or volume with a preset number of nanodevices distributed throughout the medium. A good example of this topology is in software-defined metamaterials (SDM), materials whose electromagnetic properties can be reconfigured via software [17]. This technology is enabled by a WNSN composed of identical nanodevices deployed throughout the structure of the metamaterial. The routing in this type of WNSN is based on multi-hop paths to connect two pairs of nanodevices (identified by static or dynamic addresses), which are not usually in coverage range. The principal strength of this network topology lies in the use of a single-type of nanodevice and flood-based schemes to implement nanocommunications [11]. Employing these schemes, when a nanodevice receives a packet, it checks the destination address, which is embedded in the packet. If this address does not match with the nanodevice address under consideration, then it retransmits the packet to its neighbors. However, using this communication mechanism, nanodevices must always be listening to the channel, which entails continuous energy consumption. Alternative routing schemes for mesh WNSN have been proposed [18] with features such as dynamic addresses or synchronization techniques. In comparison with those that are flood-based, these protocols optimize the retransmission process, whereas the processing complexity and memory requirement rise. Therefore, if the energy harvesting rate (i.e., how quickly the energy is gathered) is not high enough, the nanonode consumption under this type of protocols could demand the use of an external energy source. In addition, most of the energy harvested by nanodevices would be spent on retransmission tasks rather than on sending useful information.

**Infrastructure-based network**: a promising solution for WNSN that are in constant movement (e.g., a WNSN flowing through the bloodstream) is an infrastructure-based topology inspired by conventional mobile networks. In this network, nanorouters are strategically placed at fixed points of the journey. Conversely, nanonodes move through the medium under consideration. Thus, nanonodes are not permanently associated to a nanorouter, but they transmit information to the closest nanorouter in the circuit if and when they are on its coverage range, making this topology a suitable solution for body area nanonetworks (BANN), as proposed in [19]. Finally, nanorouters dispatch all the data collected from nanonodes to the gateway. From the energy point of view, the main advantage of this topology is that the largest energy consumption falls on the nanorouters, whose energy-storing capacity is larger than that in nanonodes. Moreover, since they remain static, an external energy source could easily be implemented to power them. On the other hand, as nanonodes’ energy storing is rather limited, they can stay idle while harvesting energy from the environment. When their energy level is high enough, nanonodes wake up to carry out the data transmission to the closest nanorouter by a point-to-point (single-hop) communication. If a nanonode is not within the coverage of any nanorouter, it waits until the signal can be received by one of them. Each nanonode might be identified by a unique address, which is a concern if the number of nodes is huge, due to the large number of bits required for identification.

## 3. Powering Nanodevices

**Figure 2:**
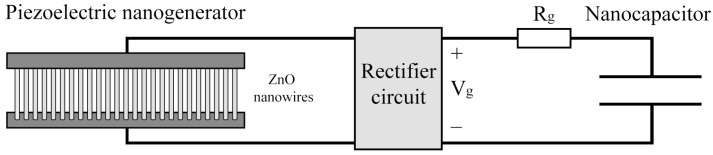
Piezoelectric powering nanosystem diagram.

Owing to the tiny size of nanodevices, manipulating or replacing a depleted battery becomes unfeasible. For this reason, in [6], the authors proposed a built-in piezoelectric generator on each nanodevice to harvest energy from the environment. This type of powering system consists of an array of zinc oxide (ZnO) nanowires, a rectifier circuit and a nanocapacitor, as shown in Figure 2.

The operation principle is simple, when the nanowires are bent or compressed, an electric current is generated between the ends of the nanowires, which is used to charge the nanocapacitor. When the nanowires are released, an electric current in the opposite direction is produced allowing the nanocapacitor to be charged once more after rectification. The compress–release cycles of nanowires are induced by an energy source, which could be either natural (e.g., the bloodstream) or artificial (e.g., ultrasound waves) [13]. This solution seems feasible, since both the piezoelectric generator and the capacitor can be miniaturized, ensuring acceptable, but still quite scarce, energy-storing capacity.

In order to illustrate the characteristics of this powering nanosystem, we analyze the results obtained in [6]. The maximum energy stored in the capacitor is 800 pJ and the time required to recharge the capacitor up to 95% of its total energy capacity is 50 s or 42 min, depending on the vibration frequency considered. In this case, two sources of energy are suggested, the vents of the air conditioning and the bloodstream, with vibration frequencies of 50 and 1 Hz, respectively. As nanodevices are self-powered and charge and discharge cycles continuously alternate, the frequency of the strains is critical to design a communication scheme, since the charging time varies dramatically depending on the particular energy source. Note that these values are for a nanodevice area of 1000 µm^2^, which is arguably significant for a nanodevice as the nature of the concept “nanodevice” might be called into question, so these values could be even more restricted.

Even though this solution has been taken as a reference in many works, it was designed for a specific communication scheme. If WNSN conditions change, that is, transmission power rises or the area of the nanodevice needs to be smaller, the design of the powering nanosystem must be adapted. For example, if 100 pJ per pulse are considered, the stored energy in this nanocapacitor is clearly too small for a feasible communication, since the number of pulses that can be transmitted without recharging the capacitor would be 8 at best. Hence, considering a piezoelectric nanogenerator as the powering component for nanodevices, we should highlight three factors that have a direct impact on its performance: (i) area, (ii) technology, and (iii) energy source.

### 3.1. Area

Each ZnO nanowire generates an electric current when it is bent, so the higher the number of ZnO nanowires, the higher the charge generated per compress–release cycle. As nanowires have similar diameter (between 50 and 150 nm), the total area of the nanogenerator (*A_ng_*) determines the number of ZnO nanowires that can be integrated. Considering the aforementioned nanogenerator, the charge generated per unit area in each cycle (Δ*Q_tech_*) is 6 fC/µm^2^, being the charge generated per cycle (Δ*Q*) directly proportional to the area of the piezoelectric nanogenerator: Δ*Q* = Δ*Qtech A_ng_*. To evaluate how *A_ng_* affects the energy-harvesting rate (*λ*), we derive its expression by modelling the generator as a voltage source (*V_g_*) along with a resistor (*R_g_*) in series, following the methodology proposed in [6]. Thus, the resistor value is *R_g_* = *V_g_/I_g_*, where *I_g_* is the average generator current per cycle. *I_g_* is defined as *I_g_* = Δ*Q f* = Δ*Qtech A_ng_ f*, where *f* is the strain frequency. Using this model, the expression that characterizes the charge stored in a capacitor as a function of the time is as follows:

(1) $$Q(t)=C_{nc}V_{g}\left(1-e^{\left(\frac{-t}{R_{g}C_{nc}}\right)}\right)=C_{nc}V_{g}\left(1-e^{\left(\frac{-t\Delta Q_{tech}A_{ng}f}{V_{g}C_{nc}}\right)}\right)$$

where *C_nc_* stands for the nanocapacitor capacitance. The energy stored in a nanocapacitor (*E_nc_*) is stated as a function of the charge (*Q*):

(2) $$E_{nc}=\frac{Q^{2}}{2C_{nc}}$$

Combining expressions (1) and (2), the energy stored in a nanocapacitor as a function of time, assuming that the capacitor is initially empty, is as follows:

(3) $$E_{nc}(t)=\frac{C_{nc}{V_{g}}^{2}}{2}{\left(1-e^{\left(\frac{-t\Delta Q_{tech}A_{ng}f}{V_{g}C_{nc}}\right)}\right)}^{2}$$

The energy harvesting rate (*λ*) is defined as the variation of energy stored in the nanocapacitor as a function of time, so we obtain its expression by means of the time derivative of *E_nc_*(*t*):

(4) $$\lambda (t)=\frac{dE_{nc}(t)}{dt}=V_{g}\Delta Q_{tech}A_{ng}f\left(1-e^{\left(\frac{-t\Delta Q_{tech}A_{ng}f}{V_{g}C_{nc}}\right)}\right)e^{\left(\frac{-t\Delta Q_{tech}A_{ng}f}{V_{g}C_{nc}}\right)}$$

**Figure 3:**
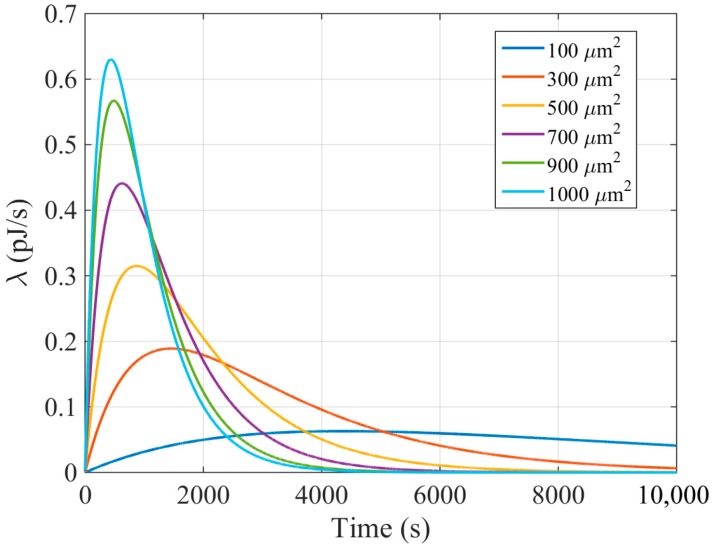
Energy-harvesting rate as a function of time for different nanogenerator areas.

Figure 3 shows *λ* as a function of time for different nanogenerator areas, with Δ*Q_tech_, f*, *V_g_*, and *C_nc_* equal to 6 fC/µm^2^, 1 Hz, 0.42 V, and 9 nF, respectively. Note that these parameters have been fixed in order to properly appreciate the impact of *A_ng_*. Even though these values could be arbitrarily set (within reasonable bounds), we prefer extracting them from an already published work to be as rigorous as possible [6]. As can be seen, when *A_g_* becomes larger, the peak of *λ* increases, leading to a quicker nanocapacitor charge. After the maximum, the value of *λ* decreases, tending to zero, indicating that the nanocapacitor is reaching its maximum storing capacity.

In turn, the area of the nanocapacitor (*A_nc_*) influences its capacitance (*C_nc_*), which is related to the maximum stored energy (*E_max_*) by the expression:

(5) $$E_{max}=\frac{{V_{g}}^{2}C_{nc}}{2}$$

where *V_g_* is the voltage generator. As *C_nc_* = *C_tech_ A_nc_*, where *C_tech_* is the capacitance per unit of area, for a fixed voltage generator, the maximum stored energy is directly proportional to the capacitance and, consequently, to the nanocapacitor area. However, as *E_max_* increases, the time to charge the nanocapacitor becomes longer, so a trade-off emerges between these two parameters. Consequently, this issue directly impacts on the achievable bitrate per nanodevice (*R*), since nanodevices must remain idle during the charging time (*t_charge_*).

To illustrate this point, we obtain the equation to calculate the time required to accumulate a given amount of energy by isolating the variable *t* from expression (3):

(6) $$t(E_{c})=-\frac{C_{tech}A_{nc}V_{g}}{\Delta Q_{tech}A_{ng}f}\ln\left(1-\sqrt{\frac{2E_{c}}{{V_{g}}^{2}C_{tech}A_{nc}}}\right)$$

Note that *C_nc_* has been expressed as a function of *A_nc_*. If *E_c_* = *E_total_* = *E_pulse_ n_pulse_* + *P_dev_ t_on_*, then this time corresponds to *t_charge_*:

(7) $$t_{charge}=-\frac{C_{tech}A_{nc}V_{g}}{\Delta Q_{tech}A_{ng}f}\ln\left(1-\sqrt{\frac{2(E_{pulse}n_{pulse}+P_{dev}t_{on})}{{V_{g}}^{2}C_{tech}A_{nc}}}\right)$$

For the sake of simplicity, since the objective is to emphasize the impact of *A_nc_* on *t_charge_* and thus the achievable bitrate per nanodevice, we assume that the capacitor is empty at the beginning of the recharge period and all the energy is consumed in the transmission. We note that due to the non-linear charging behavior of a capacitor, an optimal range of charge could be found preventing the capacitor from being fully depleted or completely charged, but the influence of *A_nc_* remains the same. Thus, as the communication starts after a period of time equal to *t_charge_*, *R* can be expressed as:

(8) $$R=\frac{n_{bits}}{t_{\mathrm{charge}}}=-\frac{n_{bits}\Delta Q_{tech}A_{ng}f}{C_{tech}A_{nc}V_{g}\ln\left(1-\sqrt{\frac{2(E_{pulse}n_{bits}p_{pulse}+P_{dev}t_{on})}{{V_{g}}^{2}C_{tech}A_{nc}}}\right)},$$

where *n_bits_* stands for the number of bits transmitted with a single charge and *n_pulse_* is expressed as a function of the probability of sending a pulse (*p_pulse_*): *n_pulse_* = *n_bits_ p_pulse_*.

**Figure 4:**
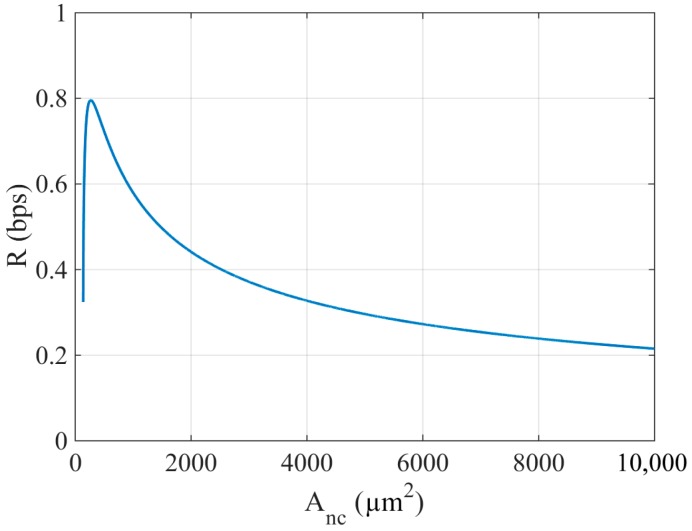
Bitrate per nanodevice as a function of the nanocapacitor area.

Figure 4 shows *R* as a function of *A_nc_* for the example given in Section 2. The values employed for *E_pulse_*, *n_bits_*, *p_pulse_*, *P_dev_*, *t_on_*, *V_g_*, *C_tech_*, *Q_tech_*, *A_ng_*, and *f* are 1 pJ, 169 bits, 0.5, 240 nW, 1 ms, 0.42 V, 9 pF/µm^2^, 6 fC/µm^2^, 1000 µm^2^, and 1 Hz respectively. The value of each one could be arbitrarily set but, maintaining the rigor of the analysis, has been taken from published works [6,13] already validated by the research community. As can be observed, for a specific application the bitrate achieved per nanodevice can be optimized by selecting the area of the nanocapacitor properly. This is due to the fact that for a given amount of energy required there is an optimal capacity that minimizes the charging time. If the capacitance becomes higher, the maximum energy stored increases, but the period of time to store this determined amount of energy (*t_charge_*) is higher than in a capacitor with a lower capacitance. This increase of time hinders the bitrate achieved by nanodevices. To the best of our knowledge, this issue has never been considered before in the related literature when designing either a nanonetwork or a nanocommunication protocol. Thus, considering a particular technology with its corresponding *C_tech_*, there is an *A_nc_* for which the bitrate is maximum. In the example above, to transmit 169 bits with one single charge, the maximum bitrate is achieved for a nanocapacitor area equal to 268 µm^2^.

Therefore, the areas of both components of the powering nanosystem are critical to further design nanocommunications, since it will influence *λ*, *E_max_* and *R*.

### 3.2. Technology

A nanogenerator based on ZnO nanowires is a promising technology for nanodevices, but its limited energy harvesting becomes evident when its area shrinks. Alternative solutions that improve the energy-harvesting efficiency should gain increasing importance to shorten the charging time of nanodevices, which is of special relevance in scenarios with weak or scarce mechanical forces. Furthermore, as mentioned before, the maximum storable energy in a nanocapacitor depends on its capacitance, which is ultimately determined by the nanocapacitor technology. For instance, Jornet and Akyildiz proposed a nanocapacitor based on onion-like carbon electrodes, which could reach a capacitance of 9 nF for an area of 1000 µm^2^, that is, *C_tech_* equal to 9 pF/µm^2^ [6]. Instead, in [20], a so-called supercapacitor is designed and fabricated with a *C_tech_* of 400 pF/µm^2^, 44 times higher than the former. This increase in capacitance means 44 times more energy-storing capacity for the same area. This is merely an example of the potential of novel capacitor technologies, which should be reviewed for future research in order to adapt communication techniques for WNSN to the energy-storing capacity available.

### 3.3. Energy Source

Two energy sources have been proposed for WNSN so far [6,21], both harnessing the piezoelectric nature of the nanogenerator, converting mechanical energy into electricity.

**Energy harvesting from the environment:** this solution is envisaged for WNSN deployed in dynamic environments, where the mechanical forces induced by the medium generate energy enough to power nanodevices. However, to the best of our knowledge, no energy model for nanodevices has studied how the energy-harvesting rate varies for different scenarios in terms of strain magnitude and frequency. In this respect, more effort is required to acquire realistic information. 

**Wireless power transfer (WPT) with ultrasound:** using WPT, the piezoelectric nanogenerator vibrates at the ultrasound frequency, generating compress–release cycles regardless of the environment. This external power supply ensures that the energy-harvesting rate is constant and irrespective of the specific environmental conditions.

**Figure 5:**
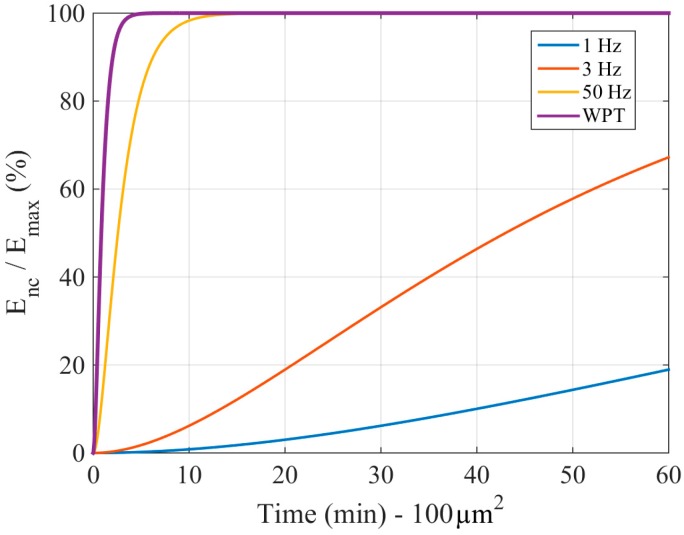
Percentage of energy stored as a function of time for different energy source frequencies.

In order to compare the energy harvested for both energy sources, Figure 5 illustrates the percentage of stored energy as a function of time for different strain frequencies and WPT, obtained from the ratio between expressions (3) and (5). Values of Δ*Q_tech_*, *A_ng_*, *V_g_*, and *C_nc_* are equal to 6 fC/µm^2^, 100 µm^2^, 0.42 V, and 9 nF, respectively. As can be seen, when applying WPT, the energy harvested per unit of time is up to three orders of magnitude higher than the energy gathered from environments with low-frequency strains such as the bloodstream, whose strain frequency fluctuates between 1 and 3 Hz.

## 4. Energy Feasibility of a WNSN

Once all the factors governing the energy management have been analyzed, in this section we study the feasibility of a WNSN from the energetic point of view, independent of the network topology employed. To this effect, we first calculate the minimum nanocapacitor area and its charging time, considering energy per pulse and number of pulses radiated as variables. Then, we derive the same area figure but this time characterizing the propagation channel by path loss and transmission distance.

Let us consider that the total energy consumption must be equal to or lower than the 95% of the total storing capacity of the nanocapacitor, defined by expression (5). This is because the time required to charge a capacitor up to its theoretical *E_max_* tends to infinite, so the upper limit of charge is set to 95%. Therefore, *E_pulse_ n_pulse_* + *P_dev_ t_on_* ≤ 0.95 *V_g_*^2^*C_nc_*/2.

Joining both expressions, the minimum area of the nanocapacitor (*A_min_*) is obtained as a function of *E_pulse_* and *n_pulse_*:

(9) $$A_{\min}=\frac{2(E_{pulse}n_{pulse}+P_{dev}t_{on})}{0.95C_{tech}{V_{g}}^{2}}$$

**Figure 6:**
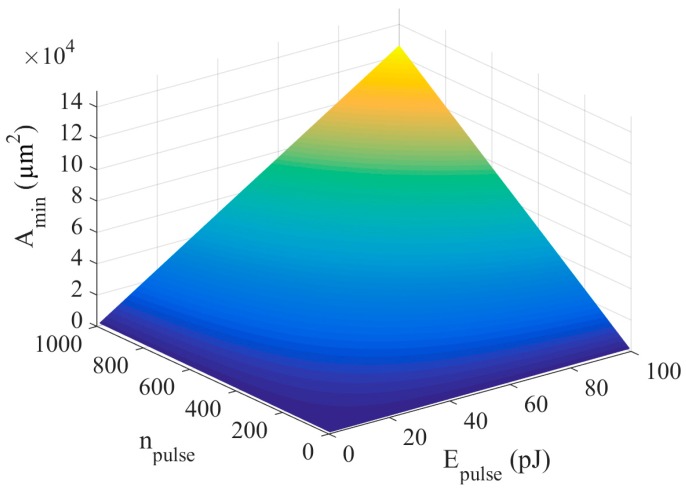
Minimum capacitor area as a function of pulse energy and number of pulses.

In Figure 6, *A_min_* is depicted as a function of *E_pulse_* and *n_pulse_*, while the remaining factors of the expression keep constant. Concretely, values considered for *P_dev_*, *t_on_*, *C_tech_*, and *V_g_* are extracted from [6,13]: 240 nW, 1 ms, 9 pF/µm^2^, and 0.42 V, respectively. As can be observed, the capacitor area required to properly power a nanodevice becomes excessive when the energy per pulse is close to 100 pJ; a usual value in nanocommunication papers. Only if a few pulses are radiated does the capacitor area remain small enough to be considered a nanodevice. 

**Figure 7:**
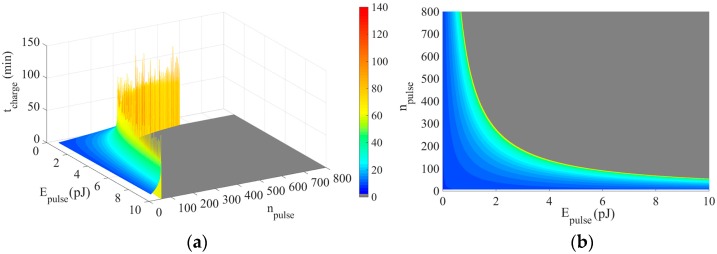
Charging time for different pulse energy and number of pulses (**a**). Same graph from top view (**b**).

We now discuss the viability of the powering nanosystem described in Section 3. Based on expression (7), Figure 7 shows *t_charge_* as a function of the energy per pulse and the number of pulses. Values employed for *P_dev_*, *t_on_*, *C_nc_*, *V_g_*, Δ*Q*, and *f* are 240 nW, 1 ms, 9 nF, 0.42 V, 6 pC, and 1 Hz, extracted from [6,13]. As can be seen, there is a delimited curve for which *t_charge_* tends to infinity, which means that the nanocapacitor is reaching its *E_max_*. Beyond this curve, *t_charge_* is equal to 0, determining the unfeasible energetic zone (in grey), that is, the powering nanosystem is not able to supply enough energy to the nanodevice. In Figure 7 (right), we clearly observe the values of *E_pulse_* and *n_pulse_*, achievable with one single charge (that corresponds to the 95% of the theoretical maximum energy stored in the capacitor, as mentioned above) for which the powering nanosystem is feasible. It is noteworthy that the number of pulses when *E_pulse_* is higher than 10 pJ becomes excessively low (<50 pulses) to perform a consistent communication scheme.

Later, we evaluate the feasibility of this powering nanosystem for different environments and transmission distances. To accomplish the communication properly, the transmission power (*P_tx_*) must satisfy 10 *log*_10_(*P_tx_*) − *L_path_ d* ≥ *S*, where *L_path_* is the path loss per unit of distance (in dB/mm) per unit of distance, *d* is the distance (in mm), and *S* is the receiver sensitivity (in dBW). *P_tx_* is set as the relation between the energy per pulse (*E_pulse_*) and its duration (*t_pulse_*): *P_tx_* = *E_pulse_*/*t_pulse_*. Considering both expressions, the minimum *E_pulse_* to fulfill the communication is:

(10) $$E_{pulse}=t_{pulse}10^{\frac{S+L_{path}d}{10}}$$

Substituting *E_pulse_* in (9), we obtain the following formula:

(11) $$A_{min}=\frac{2(t_{pulse}10^{\frac{S+L_{path}d}{10}}n_{pulse}+P_{dev}t_{on})}{0.95C_{tech}{V_{g}}^{2}}$$

**Figure 8:**
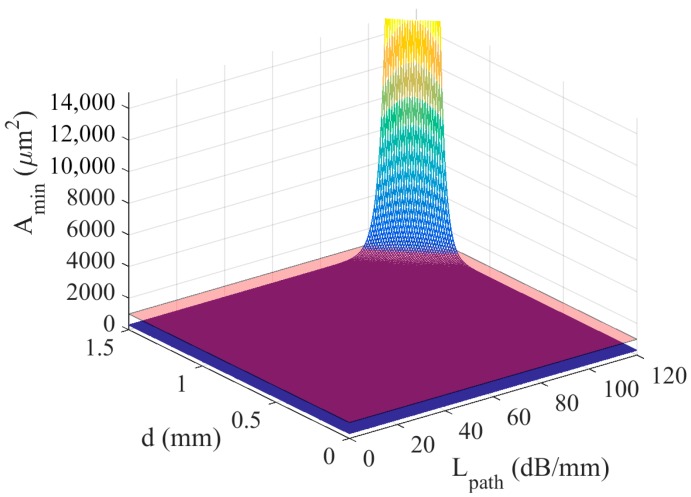
Minimum capacitor area as a function of path loss and transmission distance.

Considering *S* = −130 dBW (−100 dBm) and *n_pulse_* = 85 (i.e., enough to accomplish the blood cholesterol application proposed in a previous section), Figure 8 shows the minimum nanogenerator area (*A_min_*) in terms of path loss per unit of distance (*L_path_*) and transmission distance (*d*). Values considered for *t_pulse_*, *P_dev_*, *t_on_*, *C_tech_*, and *V_g_* are 100 fs, 240 nW, 1 ms, 9 pF/µm^2^, and 0.42 V respectively, extracted from [6,13].

Results reveal that there is a delimited zone (illustrated by a red plane) for which the nanogenerator under study is feasible from the energetic point of view. Note that this solution is unfeasible for transmission distances higher than 1.5 mm in environments with path loss greater than 100 dB/mm. Therefore, WNSN must be designed bearing in mind this hard energy restriction, since the nanocapacitor area required when high path losses become excessively high falls outside the nanodevice concept. Therefore, nanocommunications should stay within the feasibility area (in terms of path loss and distance) to be realistic and viable.

## 5. Conclusions

This paper analyzes energy management as a critical issue to be considered in future WNSN designs. We first discuss the main factors influencing the energy consumption in WNSN, highlighting the importance of the energy required for data transmission. Then, we review the most relevant parameters impacting on the functioning of a powering nanosystem, examining the most accepted solution for nanodevices, and evaluating its performance under different conditions. After a mathematical analysis, results reveal these main conclusions: (i) many already published works employed high transmission powers (even peaks of kilowatts) inadvisable for a nanogenerator with the technology proposed in [6]; (ii) the WNSN, bitrate can be optimized for a specific application by designing nanodevices with a determined nanocapacitor area; (iii) the energy harvested per unit of time by WPT is up to three orders of magnitude higher than the energy gathered from the environment; (iv) charging time remains acceptable if and when the energy required is not excessive (an unfeasible energetic zone for WNSN is defined); and (v) the solution in [6] becomes inoperative for transmission distances over 1.5 mm in high-lossy environments (exceeding 100 dB/mm), although this is the scenario usually found when applying WNSN to medicine. Regarding network topologies, more research is needed to numerically estimate and compare their impact on energy consumption. This requires further study and is, therefore, the subject of a future work.

## Figures and Tables

**Table 1:** Strengths and limitations for each network topology.

Network Topology	Strengths	Limitations
Cluster-based	- Routing complexity falls on the nanocontroller - Address reusability	- Problems if the wireless nanosensor network (WNSN) is not static
Mesh	- Simplicity using flooding - All nanodevices are identical - Able to cover a volume or surface	- Flooding implies excessive retransmissions, whereas other routing schemes are complex to implement. - Routing complexity falls on nanonodes - High energy consumption
Infrastructure-based	- Routing complexity falls on the nanorouter - Single-hop communication - Good solution for nanonode mobility	- Nanorouters (bigger in size) must be fixed over the medium - Addressing might suppose an inconvenience when each nanonode has to be identified by a unique address
